# *Burkholderia pseudomallei* Infection in US Traveler Returning from Mexico, 2014

**DOI:** 10.3201/eid2110.150815

**Published:** 2015-10

**Authors:** Jennifer W. Cheng, Mary K. Hayden, Kamaljit Singh, Ira Heimler, Jay E. Gee, Laurie Proia, Beverly E. Sha

**Affiliations:** Rush University Medical Center, Chicago, Illinois, USA (J.W. Cheng, M.K. Hayden, K. Singh, L. Proia, B.E. Sha);; Illinois Department of Public Health Laboratories, Chicago (I. Heimler);; Centers for Disease Control and Prevention, Atlanta, Georgia, USA (J.E. Gee)

**Keywords:** Burkholderia pseudomallei, bacteria, melioidosis, pneumonia, infection, traveler, Hurricane Odile, Mexico, United States

**To the Editor:** Melioidosis is an infection with clinical manifestations ranging from skin abscess to overwhelming sepsis and death. It is caused by *Burkholderia pseudomallei*, a gram-negative, saprophytic bacillus found in soil and water. Melioidosis is highly endemic to Southeast Asia and northern Australia, and endemic to the Indian subcontinent, southern China, Hong Kong, and Taiwan (*1*).

The extent of melioidosis in the Western Hemisphere is unknown. However, new endemic foci have been identified in Puerto Rico and Brazil, and sporadic cases have been reported in other parts of the Caribbean, Central America, and South America (*2**–**5*). Melioidosis is rare in the United States; 0–5 cases are reported annually, and most cases occur in travelers returning from disease-endemic areas (*2**,**3*). Case clusters have been associated with extreme weather events, such as tropical storms or heavy rainfall (*5**,**6*). We report a case of melioidosis in a returned traveler from Los Cabos, Mexico, after Hurricane Odile.

In September 2014, a 59-year-old woman came to a hospital in Chicago, Illinois, USA, with a 4-day history of right-sided upper back and anterior chest pain, fevers, and shortness of breath. She had diabetes mellitus and well-controlled HIV infection; and had received a cadaveric renal transplant 13 months earlier. Her medications included tacrolimus, prednisone, and mycophenolate. She had traveled to Los Cabos, Mexico, 7 days before admission and was present when Hurricane Odile hit the area.

On admission to the hospital, her temperature was 38.5°C, and she had right chest wall tenderness. Her leukocyte count was 22.27 × 10^3^ cells/μL. A computed tomography scan of the chest showed an irregular mass in the apical segment of the right upper lobe suggestive of a Pancoast tumor, with ground glass opacities and an enlarged right paratracheal lymph node.

She was given intravenous vancomycin, ceftriaxone, and levofloxacin. One of 2 admission blood cultures grew gram-negative rods after 30 h incubation, and antimicrobial drugs were changed to piperacillin/tazobactam. The isolate grew on blood, MacConkey, and chocolate agar when subcultured and was oxidase positive. It was susceptible to piperacillin/tazobactam, trimethoprim/sulfamethoxazole, and doxycycline but resistant to aminoglycosides and cephalosporins. Pending final identification of the bacterium, the patient was discharged on hospital day 5 and was given oral doxycycline for presumed bacteremic pneumonia.

The isolate was later identified as *B. pseudomallei* by using phenotype methods and PCR analysis at the Illinois Department of Public Health (Chicago, IL, USA) and confirmed as *B. pseudomallei* by the Centers for Disease Control and Prevention (Atlanta, GA, USA). Genetic analysis identified multilocus sequence type 92 and internal transcribed sequence type G, which is consistent with an isolate that originated in the Western Hemisphere (*7*).

Soon afterwards, the patient was readmitted with recurrent fevers and chest pain. Blood cultures were negative, and a computed tomography scan of the chest showed new partial cavitation of the right lung mass. Antimicrobial drugs were changed to intravenous meropenem, and immunosuppressive drugs were reduced. Oral doxycycline was added to meropenem after a third admission for recurrent fevers.

Although current international guidelines recommend a minimum of 10–14 days of intravenous therapy for melioidosis (intensive phase), relapse rates are high. A newer guideline recommends a longer intensive phase for some infections on the basis of results of an ongoing study in which longer courses were associated with lower relapse rates (*8*). Given this patient’s recurrent symptoms and immunosuppression, we extended her intensive treatment phase to 6 weeks, and she showed subsequent clinical improvement.

Transition to oral eradication-phase therapy was complicated by the patient’s allergy to trimethoprim/sulfamethoxazole, which is considered first-line therapy. Because treatment with oral amoxicillin/clavulanate or doxycycline has been associated with high relapse rates (*9*), we opted to give our patient combined amoxicillin/clavulanate and doxycycline to complete 6 months of antimicrobial drug therapy. She remains well 10.5 months after presentation.

Clinical diagnosis of melioidosis in nonendemic areas is challenging because signs of the disease are nonspecific and similar to those of more common diseases, such as tuberculosis. Laboratory diagnosis is also challenging. In this case, *B. pseudomallei* grew readily in culture. However, the MicroScan Walk-Away System (Beckman Coulter Inc., Brea, CA, USA) or matrix-assisted laser desorption/ionization time of flight mass spectrometry did not provide definitive species identification. For this method to be potentially useful for identification of *B. pseudomallei*, the database used would require optimization with addition of reference spectra for the organism and its close relatives (e.g., *B. thailandensis*). *B. pseudomallei*, although different from other *Burkholderia* spp. in its pathogenicity and epidemiology, is not easily discriminated from *B. thailandensis* or *B. cepacia* complex by using phenotypic tests (*10*).

In summary, infection with *B. pseudomallei* should be considered in patients with pneumonia after travel to the Baja Peninsula in Mexico, and especially after an extreme weather event. Because of risk for transmission to laboratory workers and the potential for *B. pseudomallei* to be used for bioterrorism, clinical laboratories should perform only limited work up of suspected isolates before referring them to a public health laboratory for definitive identification.
